# G-Quadruplex Structures as Epigenetic Regulatory Elements in Priming of Defense Genes upon Short-Term *Trichoderma atroviride* Inoculation in Maize

**DOI:** 10.3390/plants13202925

**Published:** 2024-10-18

**Authors:** Romina B. Agostini, Ernesto J. Piga, Candela Bayón, Andrés Binolfi, Pablo Armas, Valeria A. Campos-Bermudez, Sebastián P. Rius

**Affiliations:** 1 Centro de Estudios Fotosintéticos y Bioquímicos (CEFOBI), Consejo Nacional de Investigaciones Científicas y Técnicas (CONICET), Universidad Nacional de Rosario (UNR), Suipacha 531, Rosario 2000, Santa Fe, Argentina; rominagostini@gmail.com; 2 Instituto de Biología Molecular y Celular de Rosario (IBR), Consejo Nacional de Investigaciones Científicas y Técnicas (CONICET), Universidad Nacional de Rosario (UNR), Ocampo y Esmeralda, Rosario S200EZP, Santa Fe, Argentina; piga@ibr-conicet.gov.ar (E.J.P.); bayon@ibr-conicet.gov.ar (C.B.); binolfi@ibr-conicet.gov.ar (A.B.); armas@ibr-conicet.gov.ar (P.A.); 3 Plataforma Argentina de Biología Estructural y Metabolómica (PLABEM), Ocampo y Esmeralda, Rosario S200EZP, Santa Fe, Argentina

**Keywords:** non-canonical DNA structure, symbiosis, chromatin, priming, epigenetic

## Abstract

Symbiosis establishment between *Trichoderma atroviride* and plant roots triggers the priming of defense responses, among other effects. Currently, there is no clear evidence regarding the molecular mechanisms that allow the plant to remain alert to future stimulus, either by pathogen attack or any other abiotic stress. Epigenetic modifications have emerged as a strategy to explain the increased defense response of plants in a priming state conferred by *Trichoderma*. Recently, various non-canonical structures of nucleic acids, especially G-quadruplex structures (G-quadruplexes or G4s), have been identified as potential targets during the establishment or maintenance of plant signals. In the present study, we developed a screening test for the identification of putative G4-forming sequences (PQSs) in previously identified *Z. mays* priming genes. Bioinformatic analysis revealed the presence of PQSs in the promoter region of five essential genes playing a critical role in priming in maize. Biophysical and spectroscopy studies showed the formation of G4s by these PQSs in vitro, and ChIP assays demonstrate their formation in vivo. Therefore, G4 formation could play a role as an epigenetic regulatory mechanism involved in the long-lasting primed state in maize plants.

## 1. Introduction

Colonization of plant roots by *Trichoderma* spp. triggers an intricate molecular rearrangement of responses, resulting in higher growth and greater defensive capacity against pathogen attacks [1]. Induced systemic resistance is the result of a complex modulation of the main components associated with plant defense mechanisms. Plant immunity priming involves early signaling events induced by *Trichoderma*, which are of significant relevance. These signals enable defense responses to be activated more efficiently, rapidly, and intensely, and/or sustained over time, particularly in stressful situations [2]. Such a priming state involves a low-cost, adaptive defense reaction, since defense responses are either not fully activated or mildly and transiently triggered [3]. Defense mechanisms encompass accumulation of mitogen-activated protein kinases (MAPKs), and heightened levels of transcription factors and pattern recognition receptors, along with other proteins, hormones, and metabolites associated with defense [4]. These components can be rapidly activated in response to infection. Histone modifications also appear to play a role in defense preparation [5]. Therefore, it seems that a priming stimulus can induce accessible chromatin states for rapid expression of defense genes during coping with subsequent stress [6,7,8]. Regarding the time lapse between the two events, priming stimulus and stress are not defined and may vary among different stimuli. During this time, the defense mechanisms in question, which are often only slightly and temporarily induced by the priming stimulus, would return to almost basal levels. As a result of defense priming, a low cost–benefit balance is generated [9].

G4s are arrangements adopted by the spontaneous folding of guanine nucleotide-rich sequences in the double-stranded DNA molecule that transiently switches to a single-stranded state [10]. These structures prevail in all living organisms and play a fundamental role in their physiology. Such arrangements have gained great interest following the discovery of their involvement in fundamental cellular processes such as replication, transcription, translation, and telomere maintenance [11,12,13]. In addition, these structures have been reported to influence DNA and histone modifications, nucleosome positioning, chromatin three-dimensional organization, and post-transcriptional modulation of gene expression [14]. In relation to transcriptional regulation, it has been shown that G4 formation compensates for the local negative supercoiling resulting from the separation of strands during this process. Other studies have revealed that G4 conformations close to promoter regions can either positively or negatively affect the transcription process [15]. G4s were originally described as inhibitors of transcription, acting as obstacles for RNA polymerase [16,17]. However, more recently substantial evidence showed that G4s are mostly correlated with transcriptional activation instead of repression (although there are exceptional cases where G4s display this activity), acting in a more complex way than a simple ‘on/off’ switch [18]. G4s’ effects on transcription depend on their interaction with other biomolecules in living cells as part of an interconnected network, involving G4s as binding hubs for transcription factors and mediators of histone marks while interacting with chromatin remodeling proteins, thus shaping chromatin architecture [19,20,21,22].

Differential localization of G4s in exons, introns, and 5′ and 3′ UTRs displays completely different effects on gene expression. Particularly, G4s located in the 5′ UTR normally inhibit the initiation of translation, due to steric hindrance [22,23,24,25,26]. Formation of such structures at the boundaries between exons and introns or near the polyadenylation signals in pre-mRNAs may positively or negatively affect the binding of regulatory proteins, thus encoding different isoforms [27,28,29,30]. So far, great progress has been made in understanding the biological role of G4s primarily in humans, yeast, and microbial pathogens. However, research about G4s’ existence and their physiological role in plants is delayed compared with their study in animals (especially in humans). Recent studies have revealed that putative G4-forming DNA sequences (PQSs) are conserved in all plant species [31,32,33,34,35]. The regulatory mechanism mediated by G4s in plants is of great interest since it could provide highly relevant information for the development of improved crop varieties. In this context, recent studies suggest that G4s may be involved in regulating the expression of genes implicated in various pathophysiological conditions, including responses to biotic and abiotic stress, as well as DNA damage [31,36,37,38,39,40,41].

In the absence of reports linking G4s to *Trichoderma*-induced systemic resistance activation, we undertook this study understanding that they deserve special attention as epigenetic markers involved in the regulation of various cellular processes. For that reason, we evaluated the molecular events triggered by the priming stimulus as well as the transcriptional profile of genes of interest related to priming in maize leaves at different days post-inoculation with *T. atroviride*. Among them are the *WRKY* gene (GRMZM2G040298, WRKY transcription factor 65-related) [42,43,44,45], coding for a transcription factor potentially involved in the signal transduction downstream from the SA pathway and in signal transduction, and the *JAZ8* gene (GRMZM2G114681, jasmonate ZIM domain-containing protein) as a representative of JA pathway signaling [8,46,47,48]. Additional genes include *MAPK1* (GRMZM2G053987, mitogen-activated protein kinase/stress-activated protein kinase), involved in plant development and stress responses [49]; *AP2-EREBP* (GRMZM2G085678, APETALA2/ethylene-responsive element-binding protein), coding for a transcription factor related to ethylene response [50,51], and *ACO1* (GRMZM2G007249, 1-aminocyclopropane-1-carboxylate oxidase), linked to the synthesis of ethylene [52].

Furthermore, in an effort to unravel the events associated with this type of epigenetic regulation in priming, we assessed G4 formation in genomic DNA from maize plants inoculated with *Trichoderma*. This implies scrutinizing regulatory regions for the existence of PQSs, as well as examining their capability to form G4s both in vitro and in vivo.

## 2. Results

### 2.1. G4 Formation in Whole Genomic DNA Is Induced at Early Stages Post-Trichoderma Inoculation

**Figure 1:**
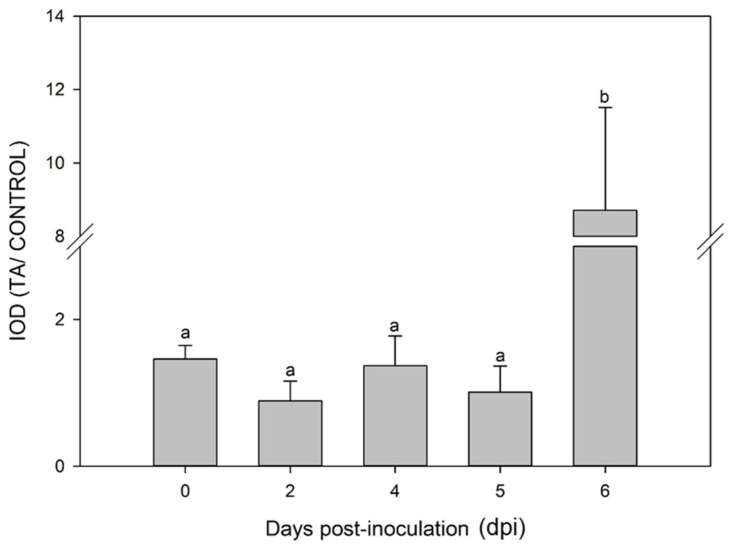
Dot-blot analysis using recombinant anti-G4 antibody (BG4). The bar graph represents the intensity ratio of optical density (IOD) of total genomic DNA from leaf samples at different days post-inoculation (dpi) with *T. atroviride* (TA) vs. control samples. Bars with differential letters indicate variations with a significance level of 5% according to the Tukey test.

Determination and quantification of G4s at the genomic DNA level was carried out to explore potential links between these and the epigenetic regulation of immune priming activated by *Trichoderma*. Dot-blot analysis of total genomic DNA samples from maize leaves at various days post-inoculation (dpi) with *Trichoderma* showed a noteworthy sevenfold increase in G4s observed specifically at 6 dpi with *T. atroviride*, as illustrated in Figure 1. On the other hand, 2, 4, and 5 dpi samples showed no significant changes compared to the control at 0 dpi.

### 2.2. Primed Genes Contain PQSs in Promoter Regions

Considering the observed surge in G4 levels in the whole genome at 6 dpi, an in-depth in silico analysis of promoter sequences from five genes associated with the priming response was conducted. The analysis was performed on proximal promoter regions (PPRs) of five genes that we have shown to be involved in the priming of the immune response: *WRKY* and *JAZ8*, as reported in Agostini et al., 2023 [8], and *MAPK1*, *AP2-EREBP*, and *ACO1* (presented in this study, Supplementary Figure S1). Our analysis using three different G4 predictors (QGRS Mapper, PQSfinder, and G4Hunter) revealed PQSs in the promoters of *JAZ8*, *MAPK1*, *AP2-EREBP*, and *ACO1* genes in the antisense DNA strand, while only *WRKY* exhibited a PQS in the sense strand (Table 1 and Table S1). Except for the *ACO1* PQS, the high scores obtained with the different predictors indicated a substantial probability of G4 formation. It is worth mentioning that the *ACO1* PQS showed score values below the threshold set by default by the predictors PQSfinder and G4Hunter, probably indicating less probability of G4 formation.

### 2.3. G4 Formation in PPRs of MAPK1, JAZ8, and AP2-EREBP Validated In Vitro

**Figure 2:**
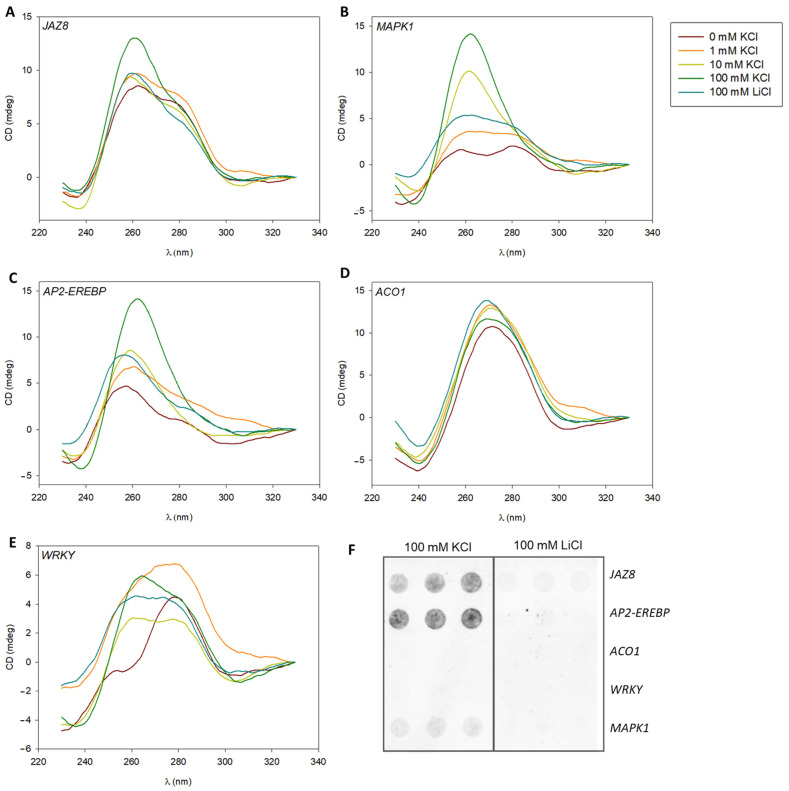
In vitro evaluation of G4 formation by CD spectroscopy and dot-blot. Oligonucleotide spectra corresponding to the PQSs identified in promoter sequences analyzed (see Supplementary Table S2). (**A**), *JAZ8*; (**B**), *MAPK*1; (**C**), *AP2-EREBP*; (**D**), *ACO1*; and (**E**), *WRKY*. The process was carried out in the presence of 0, 1, 10, and 100 mM KCl (red, orange, yellow, and green, respectively) to favor G4 formation and in the presence of 100 mM LiCl (blue), which does not favor it. (**F**), Dot-blot seeded with oligonucleotides corresponding to the analyzed PQSs at 100 mM KCl (**left**) and 100 mM LiCl (**right**).

The PQSs from the five priming genes were studied through different spectroscopic assays to assess their capability to form G4s in vitro, utilizing synthetic single-stranded DNA oligonucleotides (Supplementary Table S2). In the first place, CD spectroscopy was performed to explore the formation of G4s by the promoter PQSs for the selected maize priming genes (Figure 2). Analysis under varying concentrations of KCl or LiCl, favoring or not favoring G4 formation, respectively, revealed the in vitro formation of G4s in the PQSs corresponding to *JAZ8*, *MAPK1*, and *AP2-EREBP* promoters (see Figure 2A–C, respectively). An increase in intensity of distinctive positive and negative bands around 260–265 nm and 240–245 nm, under 100 mM KCl (in all three cases) and 10 mM KCl (for *MAPK1* and *AP2-EREBP*), respectively, indicated the K^+^-dependent formation of parallel topology G4s. Notably, the PQSs in promoters of the *ACO1* and *WRKY* genes (Figure 2D,E, respectively), did not exhibit changes in the intensity or pattern of distinctive peaks for G4 formation under varying K^+^ concentrations. However, a peak at 260–265 nm in the *WRKY* PQS at 100 mM KCl, absent at lower KCl concentrations and in 100 mM LiCl, suggests the possible presence of a G4 motif. This was subsequently confirmed at 250 mM KCl (see Supplementary Figure S2). It is worth mentioning that the *JAZ8* PQS also shows an additional increase in G4-distinctive peaks at 260–265 nm in the presence of 250 mM KCl. On the other hand, the *ACO1* PQS did not exhibit changes in CD spectrum intensity or pattern in the presence of 250 mM KCl (Supplementary Figure S2).

Verification of CD results was achieved through dot-blot experiments conducted in the presence of 100 mM KCl and 100 mM LiCl. The *JAZ8*, *AP2-EREBP*, and *MAPK1* PQSs exhibited G4 formation under 100 mM KCl (Figure 2F). Conversely, the *ACO1* and *WRKY* PQSs did not show evidence of G4 formation at 100 mM KCl. Consistently, in the presence of 100 mM LiCl, with Li^+^ being known as a non-G4 stabilizing cation, none of the oligonucleotides displayed G4 formation, matching CD results.

**Figure 3:**
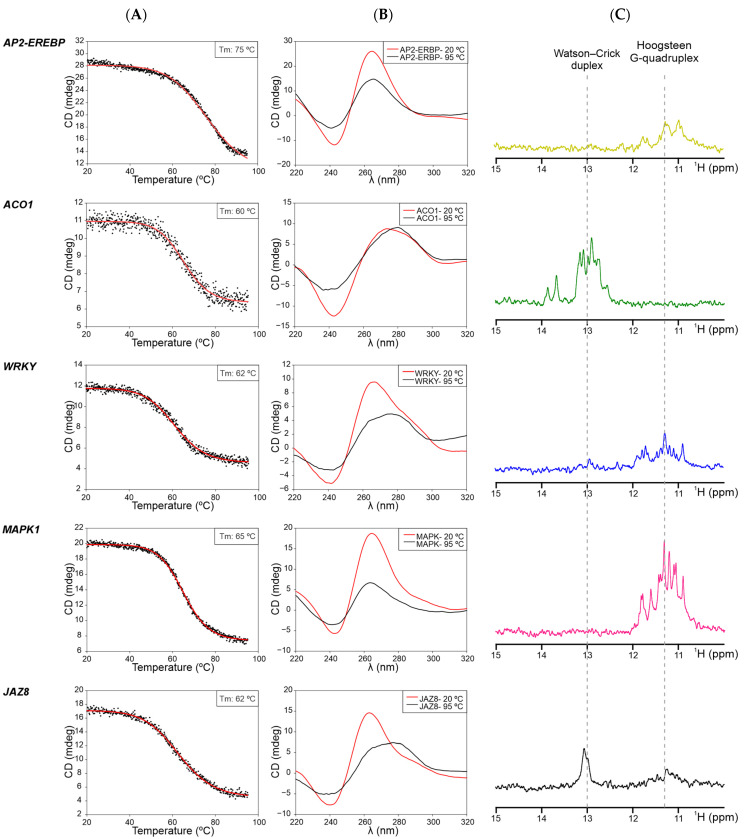
CD melting curves and NMR spectroscopy of G4s formed in vitro by the selected PQSs. (**A**), CD melting curves, experimental data (dots), and fitted curves (solid lines) are represented. K^+^ concentration was 100 mM. Estimated melting temperatures (Tm) are indicated. (**B**), CD spectra were analyzed at initial (20 °C, red) and final temperatures (95 °C, black) of the melting curve for each PQS. In some cases, the characteristic 260–265 nm positive peaks of parallel G4s are shifted to longer wavelengths as a result of G4 disassembly. (**C**), 1D ^1^H NMR spectra were obtained for each DNA sequence folded in the presence of K^+^ at the highest concentration used for CD.

To complement the structural in vitro analysis, ThT fluorescence assays and thermal melt CD spectroscopic studies were performed. The ThT fluorescence (Supplementary Figure S3) results mostly agree (mainly for *MAPK1* and *AP2-EREBP*) with the G4 formation patterns observed in CD spectroscopy and dot-blot analyses. Furthermore, CD melting curves (Figure 3A) indicated the stability of these G4s, with estimated Tm values of 62 °C (for *WRKY, JAZ8*, and *MAPK1* PQSs) and 75 °C (for the *AP2-EREBP* PQS). The CD melting curve obtained for the *ACO1* PQS may represent a melting transition of an alternative structure different from a G4, since spectra at initial (20 °C) and final temperatures (95 °C) show no G4-characteristic peaks and no clear reduction in intensity (Figure 3B).

Additionally, G4 formation was evaluated through NMR spectroscopy (Figure 3C). The presence of imino proton peaks around 10–12 ppm in the spectra of the *MAPK1, AP2-EREBP,* and *WRKY* PQSs confirmed the formation of Hoogsteen bonds and G4s. The *JAZ8* PQS exhibited low-intensity G4 signals around 10–12 ppm and coexistence with Watson–Crick structures observed as signals in the 12–14 ppm region, in agreement with the CD and ThT results described above. However, *ACO1* did not show G4 signals in the 10–12 ppm region but displayed them in the 12–14 ppm region. This indicates the absence of Hoogsteen bonds (i.e., absence of G4s) and the presence of an alternative structure formed by Watson–Crick base pairs [53], in agreement with all the results using other techniques described above.

In summary, the combined results from this set of experiments demonstrate that four of the selected PQSs indeed form G4s. Notably, *MAPK1* and *AP2-EREBP* exhibited the highest stabilities and/or tendencies to form G4s, followed by *JAZ8* and *WRKY*. On the other hand, *ACO1* proved incapable of forming a G4 motif under any of the evaluated conditions, which is consistent with the scores observed by the PQSfinder and G4Hunter G4 predictors.

### 2.4. Exploring G-Quadruplex Formation of PQSs from Priming Genes In Vivo

**Figure 4:**
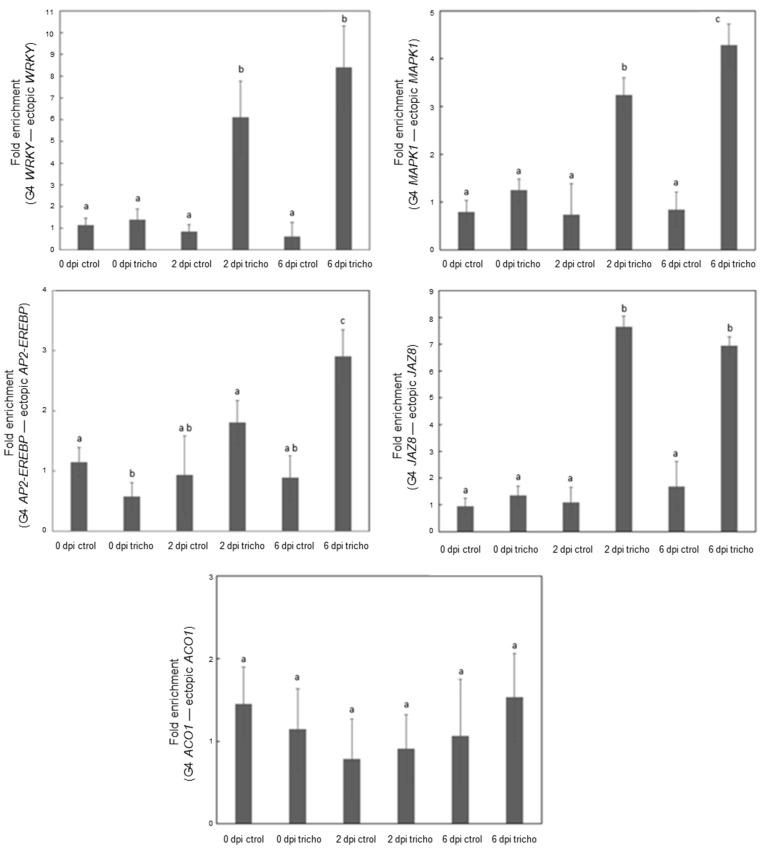
G4 presence in vivo in *Trichoderma*-inoculated maize plants. Chromatin immunoprecipitation (ChIP) experiments were conducted to quantify G4 formation in the promoter of the studied genes in maize plants inoculated with *Trichoderma*. Dark bars represent the PQS region relative to the ectopic region for each gene. Determinations were performed at 0, 2, and 6 days post-inoculation (dpi), both in the absence of (ctrol) or after inoculation with *Trichoderma* (tricho). All determinations (% INPUT) are normalized to the control condition at 0 dpi. Bars depict the mean fold-enrichment value ± standard deviation of three technical replicates under the specified conditions. Error bars represent the standard deviation. Different letters indicate significant differences (*p* < 0.05). These experiments were conducted using the third leaf of maize plants. Pairs of primers designed based on the promoters of analyzed genes are listed in Supplementary Table S3.

Further studies were carried out to determine the in vivo G4-forming capacity of PQSs present in genes involved in the early stages of priming in maize after *Trichoderma* inoculation. ChIP-qPCR assays using a specific antibody that recognizes G4s (BG4) conducted at 0, 2, and 6 dpi revealed an increase in the immunoprecipitated fraction (IP). Relevant differences were observed for *WRKY* and *JAZ8* PPRs (Figure 4), showing six- to eight- and three- to fourfold increases, respectively, at 6 dpi. In the case of *MAPK1,* a three- to fourfold increase was detected. However, the *AP2-EREBP* PQS displayed a two- to threefold increase. Furthermore, the *ACO1* PPR was not enriched by ChIP-qPCR, a result consistent with the in vitro findings.

## 3. Discussion

The priming phenomenon, characterized by a rapid and robust response upon re-exposure to a stimulus or stress, remains poorly understood at the molecular level. It is hypothesized that epigenetic modulation, which requires low energy and maintains basal gene expression levels before priming induction, could play a role [54].

Our study focused on G4 formation in maize DNA to elucidate their putative relationship with the epigenetic regulation of priming genes after *Trichoderma* inoculation. At 6 dpi with *T. atroviride*, we observed a general increase in G4s at the whole genomic DNA level. The enriched presence of PQSs in plant gene promoters suggests a family of *cis*-acting elements [55]. This finding represents the first report linking G4s with key genes involved in *Trichoderma*-induced priming. To explore regulatory mechanisms, we conducted in silico, in vitro, and in vivo analysis on five representative priming genes essential to maize defense response pathways, as previously identified [8,44]. Our goal was to investigate the relationship between their transcriptional expression response to *Trichoderma*-induced priming and the formation of G4s within their PPRs.

The in silico analysis revealed that five genes induced during early *Trichoderma* inoculation in maize possess PQSs within their PPRs. Experimental techniques, including dot-blot, CD, ThT fluorescence, CD melting, and NMR, were employed to confirm the in vitro formation of G4s in the PQSs of the *JAZ8*, *WRKY*, *AP2-EREBP*, and *MAPK1* PPRs, while the *ACO1* PQS did not fold in vitro as a G4 (Table 2).

Additionally, ChIP-qPCR assays using BG4 antibody allowed us to evaluate the presence of G4s in the analyzed promoters in vivo. The ChIP-qPCR results in Figure 4 highlight the significant enrichment of G4 structures in the promoter regions of specific priming genes, with marked increases at 2 dpi and 6 dpi, particularly for WRKY, JAZ8, and MAPK1. This enrichment at 2 dpi suggests a potential early regulatory role for G4s in the modulation of gene expression, supporting a model where G4 formation could act as an epigenetic mechanism for rapid immune priming following Trichoderma inoculation. Interestingly, while the AP2-EREBP promoter showed strong evidence of G4 formation in vitro, the in vivo results indicated only modest enrichment. This discrepancy could be attributed to the complex dynamics within the cellular environment, where chromatin structure, DNA accessibility, or interactions with regulatory proteins may influence G4 stability differently compared to isolated DNA in vitro. These observations underscore the potential differences in G4 formation between in vitro and in vivo conditions, suggesting that while in vitro assays can reveal G4-forming potential, the actual formation in vivo may depend on additional biological factors that modulate G4 stability in a context-specific manner.

In summary, after transcriptional activation, epigenetic marks would sensitize the plant to respond to infection with either faster or differential changes in gene expression. These changes would occur first, and then, as a consequence of their activation, stable epigenetic marks (such as G4 formation, among other possibilities) could be added, resulting in genes ready to respond quickly to a new stimulus. Thus, our results may further increase current understanding of the early activation of priming genes induced by *T. atroviride* in maize leaves. Although more questions than answers persist regarding the epigenetic regulation of plant immune priming, our findings shed light on the direct activation of genes related to different pathways of the early priming response after inoculation, in a process involving G4 formation. While our findings reveal the presence of G4s in maize DNA and suggest an association in the regulation of priming genes by *Trichoderma*, further studies are required to establish their role in plant immune priming and epigenetic regulation, as well as to address several questions: (1) regulatory mechanisms underlying the process, (2) stability of G4s during priming, and (3) G4 parent-to-offspring transmission. Additionally, exploring interactions between G4s and epigenetic marks during *Trichoderma*-triggered priming in maize is essential.

Given the importance of plants in domains such as food, clothing, medicine, and energy, coupled with the potential role of G4s in coordinating gene regulation, research on plant G4s shows promise for applications in plant improvement strategies.

## 4. Materials and Methods

### 4.1. Fungal Strain and Plant Material

*T. atroviride* IMI206040 and *Z. mays* seeds (line B73) were used. Strain growth and seedling inoculation are described in Agostini et al. (2023) [6]. Briefly, 10 days after germinating, seeds were inoculated with 1 mL of a *T. atroviride* conidium suspension (1 × 10^5^ conidia.mL^−1^), while the control samples were inoculated with water. Subsequently, the third leaf of each plant was collected at different times—0, 2, 4, 5, and 6 days post-inoculation (dpi)—and stored at −80 °C. Each sample was prepared using a pool of maize leaves corresponding to each experimental unit. This protocol was carried out in triplicate, where the experimental unit consisted of three independent plants.

### 4.2. DNA Extraction

Leaves (100 mg) from the *Trichoderma* inoculation experiment at different times were used. Tissue was pulverized in a mortar with liquid N_2_, and then 0.6 mL of extraction buffer (1.4 M NaCl; 100 mM Tris-HCl pH 8.0; 20 mM EDTA; 2% (*w*/*v*) Cetyl Trimethyl Ammonium Bromide) was added. The mixture was subsequently incubated at 65 °C for 15 min followed by addition of 0.2 mL of a mixture of chloroform/isoamyl alcohol (24:1). The DNA pellet was resuspended and treated with RNAse A (TransGen Biotech, Beijing, China). DNA concentration was determined spectrophotometrically at 260 nm (Epoch 2 Microplate Spectrophotometer, BioTek, Winooski, VT, USA) and integrity evaluated by electrophoresis on a 1% (*w*/*v*) agarose gel.

### 4.3. Evaluation and Quantification of G4s

In silico analysis: Prediction of the presence of PQSs in the promoter of the genes under study was carried out using the sequences corresponding to 1000 nucleotides (nt) upstream from the transcription start site (TSS) and the sequence corresponding to the 5′UTR, defined here as proximal promoter regions (PPRs). Three PQS predictors were used: (1) QGRS Mapper software (https://bioinformatics.ramapo.edu/QGRS/index.php, accessed on 25 March 2024) [3,56]: in this case, parameters were set as follows: max length: 45; min G-group: 3; loop size: 1 to 15. (2) PQSfinder web (https://pqsfinder.fi.muni.cz/, accessed on 25 March 2024) [57], with parameters set as follows: maximum length: 45; minimum score: 47 (by default); allowed defects: 0; minimum loop length: 1; maximum loop length: 15; allowed bulges: 0; allowed mismatches: 0; minimum G-run length: 3; maximum G-run length: 11 (by default). (3) G4Hunter web (https://bioinformatics.ibp.cz/#/analyse/quadruplex, accessed on 25 March 2024) [58], with the following settings: threshold: 1.2 (by default); window size: adjusted by PQS length.

In vitro analysis: Synthetic single-stranded DNA oligonucleotides were used to assess G4 formation. Oligonucleotides were designed to contain the complete selected PQSs flanked at both the 5′ and 3′ ends by five additional nucleotides corresponding to the reference sequences informed in the reference maize genome. Synthetic single-stranded oligodeoxyribonucleotides (Supplementary Table S2) were purchased from Macrogen Inc. with cartridge purification, dissolved in double-distilled water and stored at −20 °C until use. Concentrations were determined by spectrophotometry using extinction coefficients provided by the manufacturer.

(1) Circular Dichroism (CD) spectroscopy: Oligonucleotides were subjected to folding reactions to form G4 structures. Reactions were performed by denaturation of oligonucleotides (2 μM) in 10 mM Tris-HCl buffer, pH 7.5, at 95 °C for 5 min and subsequent renaturation by slow cooling until reaching 25 °C. This process was carried out in the presence of KCl (0.1, 10, 100 mM) to favor G4 formation and in the presence of 100 mM LiCl to prevent it (negative control or no G4 formation condition). CD spectra were recorded on a spectropolarimeter (Jasco J-810), using a masked quartz cuvette with a 1 cm path-length, scanning speed of 100 nm/min, continuous scanning mode, measuring ellipticity every 1 nm, time response of 1 s, bandwidth of 1 nm, sensitivity of 100 mdeg, and 230–330 nm wavelength range. The spectrum obtained for each sample represents the average of four spectra recorded consecutively and corrected according to the corresponding baselines and blanks (Spectra Manager software Version 1.53.01). The signal-to-noise ratio was improved using a Savitzky–Golay digital filter with a convolution width of 25.

CD melting experiments were performed as described elsewhere [59]. Briefly, melting curves were recorded by ellipticity measurements between 20 °C and 95 °C at the wavelength corresponding to the maximum for the positive band around 260–265 nm. The ellipticity response to temperature was analyzed using a nonlinear fitting equation assuming a two-state transition of a monomer from a folded (G4) to an unfolded state. The reported Tm represents the temperature at which both states are equally populated.

(2) Determination of G4s in vitro by dot-blot: To perform the dot-blot assay, samples (100 μL) containing 2 μg of total genomic DNA were loaded onto a nylon membrane (Amersham Hybond^TM^ N+) in a Manifold-I Dot-Blot System (Schleicher & Schuell) in triplicate. After loading, each well was washed with 0.3 M NaOH (200 μL). The membrane was then baked for 2 h at 80 °C. Immunodetection of G4s was accomplished with recombinant anti-G4 antibody (BG4) fused to a FLAG-tag sequence [58], which specifically recognizes G4s. Then, the membrane was incubated with anti-FLAG antibodies (Sigma-Aldrich Cat. No. F3165). Subsequently, the membrane was incubated with anti-MOUSE antibodies conjugated with the enzyme horseradish peroxidase (HRP) (Jackson Cat. No. 115-035-003). Finally, peroxidase activity was revealed by chemiluminescence (Bio-Lumina detection kit, Kalium Technologies). Real-time chemiluminescence was detected using Amersham^TM^ Imager 600 equipment. The spots were quantified using Gel-Pro Analyzer 3.0 Software.

Dot-blot assays to detect G4 formation in DNA oligonucleotides using recombinant anti-G4 antibody (BG4) [60] were performed as previously reported [61]. Assayed DNA oligonucleotides were previously folded in the presence of 100 mM KCl or 100 mM LiCl.

(3) ThT fluorescence assays: Thioflavin T or ThT (3,6-Dimethyl-2-(4-dimethylaminophenyl) benzothiazolium cation, Sigma-Aldrich T3516) fluorescence assays were performed as previously described [21] using a final concentration of 1 μM DNA oligonucleotides folded in 10 mM Tris-HCl buffer, pH 7.5, supplemented with 100 mM KCl. A threshold of fivefold increase was used for considering G4 formation.
(4) 1D ^1^H Nuclear Magnetic Resonance (NMR): NMR spectra were registered at 20 °C on a 700 MHz Bruker Avance III spectrometer (Bruker Biospin, Andover, MA, USA) using a triple resonance inverse NMR probe (5 mm ^1^H/D-^13^C/^15^N TXI). Oligonucleotide samples (50 µM) were loaded into 5 mm Shigemi tubes (Shigemi Co., Tokyo, Japan). We employed a pulse program incorporating water suppression [62], with NMR parameters set as follows: 8 K points, 1024 scans, a recycling delay of 1.4 s, and a sweep width of 22 ppm. This resulted in an experimental time of 29 min. Data processing was performed with an exponential multiplication (LB 10 Hz) followed by baseline correction. All spectra were acquired and processed using Topspin 3.5 software (Bruker, Biospin, Andover, MA, USA).
(5) In vivo analysis: G4-chromatin immunoprecipitation (G4-ChIP) ChIP-qPCR experiments used a classical protocol [63] with few modifications. Recombinant anti-G4 antibody (BG4) and Anti-FLAG^®^ M2 magnetic beads (Cat. No.M8823, Sigma-Aldrich, Saint Louis, MO, USA) were used. Specific *JAZ8*, *WRKY*, *MAPK1*, *ACO1*, and *AP2-EREBP* regions were amplified by qPCR using primers listed in Supplementary Table S3. A pair of primers were designed to flank PQSs and a no-G4 region (ectopic region) more than 500 bp upstream from the PQSs of each gene. The 2^−ΔΔCt^ method was used to determine G4 content as indicated using in silico predicted PQSs. Ectopic regions were used as a negative control. INPUT was used as a total DNA control.

### 4.4. Statistical Analysis

The statistical comparison and degree of significance of the differences analyzed were obtained by using the variance analysis test (ANOVA) with one or two tails. Multiple comparison analyses were performed with Tukey’s test. In all cases, the confidence level used was 95% (*p*-value < 0.05). These analyses were performed using GraphPad Prism v7.0. Quantitative data are presented as the mean ± SEM (standard error of the mean).

## Figures and Tables

**Table 1:** Prediction of G-rich sequences forming G4s in the promoters of genes under study. The results of the approach with a minimum of 3 guanine nucleotides per tract are represented. The table indicates the gene under study, type of DNA strand (sense or antisense), PQSs, length, and scores of the PQSs found in the three G4 predictors. Scores marked by * indicate values below the default threshold set by the predictors. G nucleotides probably involved in G4 formation (forming G tracts) are underlined.

Gene	DNA Strand	PQSs	Length	Predictor Scores		
				QGRS Mapper	PQSfinder	G4Hunter
*JAZ8*	Antisense	GGGTGGGGCCTTGGGATTCTCCGCGCGGGGAACGGG	36	65	54	1.361
*WRKY*	Sense	GGGATGGATGGGGATCTGCCGGGCGGG	27	66	56	1.556
*MAPK1*	Antisense	GGGGACTGGGGGACGGGCGGG	21	69	66	2.429
*ACO1*	Antisense	GGGCATTGACCTGTGGGCACCCACGCGGGCCACGCCCATGTGGG	44	69	36 *	0.227 *
*AP2-EREBP*	Antisense	GGGGAGGGGCAACTGAAGGGGGGG	24	65	61	2.458

**Table 2:** Summary of G4 formation evidence by the PQSs in the PPRs of analyzed genes. The plus (+) sign represents a qualitative estimation of the evidence of G4 formation observed with each technique.

Gene	Dot-Blot BG4	CD	ThT	NMR	G4-ChIP
*MAPK1*	+	++++	++	++++	(+2, ++6)
*AP2-EREBP*	+++	+++	+++	+++	(+2, +6)
*WRKY*	-	+	++	+++	(++2, +++6)
*JAZ8*	+++	+	+	+	(++2, ++6)
*ACO1*	-	-	-	-	-

## Data Availability

Data is contained within the article or supplementary material.

## Supplementary Materials

The following supporting information can be downloaded at: https://www.mdpi.com/article/10.3390/plants13202925/s1.
