# Supplementary material for: G-Quadruplex Structures as Epigenetic Regulatory Elements in Priming of Defense Genes upon Short-Term Trichoderma atroviride Inoculation in Maize
Source: Plants (Basel). 2024 Oct 18;13(20):2925. doi: 10.3390/plants13202925 (PMC11510774; doi:10.3390/plants13202925)
Supplement: Supplementary file 1 [file plants-13-02925-s001.zip › plants-3199300-supplementary.pdf]

Supplementary Table S1. Sequences of the five analyzed PPRs and their predicted PQSs. Sequences of the five analyzed PPRs are represented in black color letters, while the sequence representing the 5'UTRs are represented in red color letters (first exon) followed by black color letters (second exon). PQSs found in the sense (coding) DNA strand regarding transcription are highlighted in yellow. Sequences complementary to the PQSs found in the antisense (template) DNA strand regarding transcription are highlighted in yellow. G nucleotides probably involved in G4 structures (forming G tracts) of PQSs found in the sense (coding) DNA strand are shown in bold and underlined. In the case of PQSs found in the antisense (template) DNA strand, C nucleotides complementary to the G nucleotides probably involved in G4 structure (forming G tracts) are shown in bold and underlined.

Gene	Gene ID	PPR sequence and PQS found
JAZ8	GRMZM2G114681	CAATTTTAGTCAAAAGTTATAAATTTTGCTAAATTTATATAATAA<br>AATAATATTATAGTACTAAATAAGTATTTCTTTAATTATATTTCT<br>ACAATATAACTATTTAATATTGCAACTTTTGTTATTATTTTTTAC<br>AAATTTAATAAAAGTTAATACTTGTTTTGACTATTGGTGAAAGTT<br>AAAATGACTAAGGAAAGAAGTAGTAGTAAAAGAGAAGCGTCGTGC<br>GCACATGGCACAGGTGATGGCTAGAAACCATAAACAAAAACCGTC<br>GCGGCACGCCACCCGCTAAAGTCCGAAGCCCCCGTGACACAATTC<br>CTTTACCTAGGAGTACACATATTATTTATTTATTACTTGTTTACT<br>TTTTTTTTATTTAGTGCCTTTTGTAGCTTTCCCCCCGACACCAAC<br>TGACCTGATTCAATATTTCATCGGCACAGTGGGCCCAACCGCGCT<br>GCGCCCATCGTGGTCCGAACAGGAACCACGTGGGA**CCC**GTT**CCCC**<br>GCGCGGAGAAT**CCC**AAGG**CCC**CA**CCC**GCCACCACCGCGCCAGTCG<br>CCTCGTGCGCTCCCCGTCCCGAACACGTGTCGTCCTATCGATGAC<br>CCGCACACCGGCTCCGTGGCACGGTGGACGCTCGGCCGCCGCAAA<br>CCCTTCCACCGACATGCGGGTTCCGTCCATCTCTGCCCCAGATGT<br>CAGTGACGTGCACGGGACTCACCGCGGTGCAGCGGGAAGGGCGTC<br>GTGCCGTACCACGTGGGGGACTCGACCGGGGAACCGATAAACGTG<br>GCGCGGGCCCCGCCGAGAGAGACGCGCCCGATCCGGTCACCGCGG<br>CTCGGGCCCGCCGCCTCTAGTCGCCAGTCGCCATGCGGACCACTC<br>AATTATCTCCTCCGTTTCCTTAGCCATTGTCAGCTGAGTAATCTA<br>ATACCCTCGTTTCCAATTAAGAAAGGATCGCAGGCCATTAAACAA<br>GCACACACGGGAGGCCTACAAAGACGAGAGGGGAGAGGAGAACGG<br>TCAAGGCTGCCGCGCGCATCGTCATCAGTTCACTAGTCAGTCGAG<br>GTCGAGGGGCGGACGAGGAGCTGAGGAGAGAGAAGCTTCAAGGAG<br>TTGGGGACTTGGGGGTTGCCGAGCTGTCCGTCTTTCCCCTCCCCT<br>CCACCCACCAGCTACCTCGGGAGGGCAAGGCAAACATCTGCGGCC<br>TCCTTTCCCGAACCCGGGTTGG
WRKY	GRMZM2G040298	TGCCATAGCTGAACCCCCAATTGAATAATAATCGCGTGAGAGCAC<br>GATCGAAGGAGTCGCCAATGCAAAAGAAGAAGAAAAAACTATGGC<br>TTCGTGTTGCGGTGGATGTATGGAACGCGTGATGTCTATTTCCAT<br>CCAAAGAATCTGCGGTTGGATTATTGGATCTGTGTCGAACACACC<br>AAGCCGCTGCTAAGCAATATATATGACAGCGTCAAAAGGTTAATT<br>TGACCCAGGCCAGGATATCATATCATAACATATCATATCTAGAAT<br>AGAAACTAGACTCACTCTACTAGAAGAAGAAGAAGAAGCAATGTA<br>AAATTCTGAAGCTGGTAGCGACGTATATCGTTGATTCTTCACCAA<br>GCAACCGAATACGACGACAAGTCACGACCTGAATTACCACGGCCT<br>TTACTCCTGTCTCCTAGCCTGACGTGTCTGCCGAAATATCCGACG<br>CCTCTAGCTAGATTCTTTTTTATCGGAGTAGGCGCGGGCAGAAGA<br>ATTCCGCCCGGCGCGCGTGTGCAGCTGTCAACGTTTAACCCCTCG<br>ATAGAAACGCGCGT**GGG**ATGGATG**GGG**ATCTGCC**GGG**C**GGG**CTCC<br>GCCGTAGTCCGGGACGAGCGGCCACTGCACTGCCATGCACGCACG<br>TCGCAGTTCCAGGGCACTGTTGCCGCGCGTGGGCATGACGGCGAA<br>CCGAGCTCTGCTGCTGGTTTGGTTCTCGACTCCAACCGAAACCAC<br>GCACCAACCACTCCAATGCATGATGATATGCGTACGTTAATAATT<br>AATGCATATGCTACTTCTCATCTAATTCCCGAATCCCGAACGAAC<br>GATGACAGATGAGGGGACGACGACGATCCATCATCAAAGGCATGG<br>ATGGATGGATGGACATGGCGATAGTTGGGGAGACGTCGCCGTCGC<br>ACGCTCGTCCTCGTCTTCTCAAGAGAGCAGCTTGTCTGGCTGTCT<br>CTCCTCTCCTCTTTGTTTAAGTTCGTCCCCCGCGCGCGGTCTCCA<br>CGCCTTGCTCAGCCTCAGCCCTCAGGCCTCAGCCAGCGTGCGCGC<br>CCACAACCCCTCACGTCGTCCCCGCCAATCACACCGCACGAACCG<br>AGGACGACGGGACACCACCGTCACCGCGACGTAGTAGTAGTACTA<br>CATGCATAGCATAGCCTCGTGGAGTCGTGGTAGTAGTAGTTTAGT<br>TTTGCTCGCTTCCACACCGCACCGCCGTTCCCTTTTTTATATCCC<br>GCGGATGCGCCTGCCTCTGCCTCGGGGTCGGGGGGCAGCTGGTG<br>ACATCGGATTCGGATCAGGCCGAGAAGATCGAACTGCTCCGATCC<br>GGCGCTTAATTTCTCTCTCTCTCTCTCGGGCTAGCTGCCCGGGCA<br>A
MAPK1	GRMZM2G053987	CACAAACAATGGCCTATCTCTCTCTGACTCCCATCAGCTCGTCGA<br>GCCTAGTGACGGCCCCACGTCTTGATCTGAGAACCGTACGGAATG

		ATACTCGCCGCCAAAAGAAAATTGCGAAACCATAGATAGGATGGC<br>CGTAACTTCAGCAATCTGTCTCTCCCACACAGAAAGATGGAGACG<br>TGCAAAATAAATAGGAATCCGGAGATACTTTGCTCAGCTCGCGTA<br>TCACGCGGGAACAATAGAAAGTTGGGCTTCGCAATCATTCATGGC<br>ATCGGTTTCTTAAGCCCTCCCTTGTTCCAATCGCCCGGGGCAACA<br>CTGTACTGCTAGCAGTACACATCAGTTTGTTATTAGGGGTTCCTC<br>AATCCTCATCCATTAGGATATTTTCCTGACAAAAAAGAAAAAGAA<br>AAGAATGGGCACTTGCACTGGAAACTAGTTTCAGTTCTGCATCGA<br>GCCGCCAAGCATCGACCTTGTCTTTTGCAGGGGCCAGTGCTTGTG<br>CTTGTCGACCAATAGTGAAACTGGATTCGTTAAACAAATCTTGAG<br>CATATATATTGAATGTGTCGGATCTGTTTAGAGACGCCGGGACCG<br>TCCCAAATCTGCCTGCAGGAGGCATTCGGGAACATTGGGGCTGTA<br>CCGGACATCTTGAAATCCTTGTTGTATAGTTTTCTTTTTGAGAGA<br>GCAGCCGGAAGAAGATACACCACGTGATCACACCACACTACTAAG<br>TCAAGTTACACTTGAGCCTTCTCACTGCTGAAGTTTAGCACTTAC<br>CTCCGGACCGCGGCCGATGTCAACAACCTATAATAGGCCATAGCT<br>TGGAGTCGGGAGTCGGGACTCGGCTTTCCACACGTTGCCACTGAG<br>CCACTGAGGACGACGACACGGGCGGTCAAAAGCCACCGCGGCAGG<br>CACGGCCAGCCACGCACGCAGACCGCATCATCAGCCATGGCCCAA<br>CGCCCAAGCGCCGCAGACCGGACGTCATTCCCTGACGCGTCCCCG<br>TCCAATCCGGCGCCGGCGCCAGCAAAATCAACGCGTCGGCGGCTA<br>TCGGCCAATTGCCAATTCCTCCCGCCGAACGCTGAGACCCTGACA<br>CGGTGGCCCACCATCGCAGCAGGGCACCCCTGCCTCCTCCTCCTC<br>CACTCACCAGCCCAACCGCGTGCGACGTCCGTTATCCGATCTGGT<br>GGGTGCGTGTCCTCGTTTGACCGTTGAAGAGCCCTGCCCGCCGTC<br>GGATCCGAACAGCTTCCCTTCCATCAGATTCAGAGCGCCGTCCGC<br>AGCCGACTAGCAACAGTGGCCGTCCCGCTCACTTTCTCGCTACCT<br>TCCTCAGCCGGAGTCGTGGGCAGAGCAGAGCGATCGAGGCCTGCT<br>TCAGCAGTTCAGCTGCAGCCGCCG**CCC**G**CCC**GTCC**CCC**AGTC**CCC**<br>ACCTTCTTTTATAAATCCCACGCCTGCGCTTCCTCTTTTGACGAA<br>TCGGTCGCGAGGGGAGAAAGATAGGAGTCAATTAATTTGCCTGTT<br>CTGCTTGCTGCTGACCTTGTGAGTGAGCCGCCGGTCTTCCCCTTC<br>CTCGCCGAGGGGAAATAATAAGGCAGCCACAGCCAGGGCCGCCCC<br>GAGGAAGAAG
ACO1	GRMZM2G007249	GATTCACTGTCATATGATGAACTAGTTGACTTGCTAAAAGAATAT<br>ACTCAAAGTCATTAGAAAGAAAAAAAAAATAAATGTGACAAGTTA<br>AAAACTAAGAATAAAGCTATGTTTGGAACCTTAGAGCTAATAGTT<br>AGCTAGCTAAGAAATTGTTAGATGGGTTGAGTCAGTTAATGGACT<br>AATTGTTAGCTTTGAGTTAGCTAACAATTAGATGAGGCATTAGCT<br>AGGGGTCTAGCTATTAGTTCTAGAGTATTCAAACAGAACCTAAGT<br>CTTTATATGATAGGTGTTACATAGACATAAAAATTAATGATAAAG<br>TAAGATATGAAAATCAAGCTATGTCGTCCAATGCTAAAGAGTTTA<br>AATCTTCTCTTAGAGATATAAAAGAAAAATATGATAAACTTGATA<br>AAAACAAACGAAGAGTTTATGATATATTTGTTTTTTAAAAATTGA<br>CCGTGACAATATTGTTGCTGCTTATAACACATGATACTACTAACA<br>AAAGAGTTTTCATATAACACATTATTACCAACAGATATCATCGGC<br>GTCTCATTCCTTTGCAGACGCTCAGACGCCCGGGTGGGCGGTGGC<br>GGCACGGCGGTCCCGCGATCGAGACGGTTTGCCATGGGCCGCTCA<br>ACCTTCGCCACGGAGACAAAATGGCATTTGCCATACTGTGCCATA<br>TGGCTTGCCATCCCCAACTTGAGTGCTCGACCACGGAAGCGCACC<br>TCACCCCATCCATCCGCCACTGTCGACGGCTGCCCTTCCCCGAGC<br>TGACGGCCAGCCCAGCCAAGCCAAGCTGGAGTGCAAGAGAATCCC<br>GTGCGTGCATGCTGAGGGCCCGCGACGAGACGGGCCAACACGCGT<br>CG**CCC**ACATGGGCGTGG**CCC**GCGTGGGTG**CCC**ACAGGTCAATG**CC**<br>**C**TGTCTGTCAGCAAGAGCAACAACCAAAAAACAACTCTGCTGCTG<br>GCTGCTGTCTGTTGACAAGTCGGGAAAGCTCGTCCACTTCCAGTT<br>CCACTCCGCTAGAAAGCTTGAACTTGGATGCCGAGCCTATAAATG<br>GCGACCGACCCCGGCCACTTCCACTCACCGCACTCCAGCGTTCAG<br>CATTAGACACGAGAGCTCCTAGTAGCCAGACCAGTAGTCCCGCGA<br>CCCTGTCGAGAGAAACAGACAGAGCAAC
AP2-EREBP	GRMZM2G085678	TTCCAAACAAAATGGCAACAGTTTTTTTTTCTGGAAAGCACCCAA<br>ACGGGAAAGTTGTGGGAGAGGGAACACAACATTTAAGTGTGCAAA

		GTCGTAATCTGCAACCAAACATTTTCAGCAAAGACTCCAGCTCCC<br>TTTCAAAGAACCCAAAATTATTAGAATGTTTGGATACAGCGAAGG<br>ATCTGATCTTACTATAAACATTTGTCATCCTAAGACAAGAGTAAG<br>CTTATTGCTGAAGCAGACTACCATAAACCTTTAGAGCTACCGTCT<br>TGTGGATAGTTTCAGAGGTTAACGAAAGAAAGCCAAGTTCAAGTG<br>AAATGAAATGAAATTTTCTACAAACCGAAGCACACGAGATGTTCA<br>ATGGAGAAGAAATGAAGTTACGTGTAGACAGATTATTATAGCCTA<br>ACTTCATAACCATTTTGCAACCTGATTTCTATTGTGAGAATATAG<br>CGAAGCCAAAATGTAAACATACTATCAAATTCTCCACTGAACAAC<br>AGTTCAGTCTACTCCCATATTATAAGTGACAGAAAAAAAAAACTT<br>TTATATTTCTTCATTGAACAAGTATCCATCAACTGAGCACACACC<br>ATCATCATCACCAGTCTGTACCTGCAGCTACCTCAGACAAGCTTA<br>AATGCCAACCATCTCCAAGCAATTCCAGAACTAACACCCCAGGAC<br>AAAGGCACCCTTGTCAATTCTCATACACAGGTCGCACTGAAATCA<br>CTCTAAACACACAATCTATATTCAGTTCAAGAACAATTCACTGTA<br>AATTTCAACATCCCATTACATATTTATAGAGAAAAAATTGGGTGA<br>CGGAACACGACTAGAGATTCCAAATGAAGCAAAAGAATTTTGACG<br>TTATCTCAAAAATATGCAGTAAATATTCCAACACCCTAAGTTTCA<br>GCACAAGTCTTACCTATATGCTTTGCCGCAGTGTCCACGCTTCCA<br>GTGATACTCAACAATATGATTGGCCCCAGTAGCTCTGGAAAAAGA<br>GATGAAGTTATCCAAAACAGATGTACATTAACCCTACATAAACTA<br>CTACTAGGACTATGTGGATCAACTTAGGGTTGCCCCACAAATAAA<br>TGTGGCAGCCATGGGTGTAGGTGAGAGTGTTTATTACCACTGTAC<br>TAGCAGGGAAGGTGGTTATGCTATGCATACACACAGTTCACCGAC<br>AGCCCACACCTGCAGACACCATGTGG**CCC**C**CCC**TTCAGTTG**CCC**C<br>TC**CCC**TCATCTTTTGCCTCTATAGTTTTATCCATTGACGCACGCT<br>TGGTCTACTGCTCTTGGCCCCAGCACCCCTGCTCTCCAGTCAGTG<br>TATGCCGCCTTCTATCAAGGTGGCTTGGTGACTGGTGAGTGAAAA<br>CTAGGTCCAGACATATTCTCCACTCCTTTCCTCTCTTACTACCTA<br>CGCAGCTAACCACATTTATCACTCACTGCAGAAGCAAGTACAGGC<br>TGGGCAGTTTATATTCACTCAAACCAGCCCGTTCCTCCCCCACAC<br>GGCCACACACACTTCACTTCATAGGCGCCCCAGCAGAGCTCATCT<br>CTGCCTCCTGTGTCCTTCTCC

Supplementary Table S2. Oligonucleotide sequences to evaluate G4 structures *in vitro.* Oligonucleotides were designed to contain the complete selected PQSs flanked both at 5' and 3' ends by five additional nucleotides corresponding to the reference sequences informed in the reference maize genome.

**Gene**	**Gene ID**	**Oligonucleotides**
JAZ8	GRMZM2G114681	GTGGC-GGGTGGGGCCTTGGGATTCTCCGCGCGGGGAACGGG-TCCCA
WRKY	GRMZM2G040298	CGCGT-GGGATGGATGGGGATCTGCCGGGCGGG-CTCCG
MAPK1	GRMZM2G053987	AAGGT-GGGGACTGGGGGACGGGCGGG-CGGCG
ACO1	GRMZM2G007249	AGACA-GGGCATTGACCTGTGGGCACCCACGCGGGCCACGCCCATGTGGG-CGACG
AP2-EREBP	GRMZM2G085678	GATGA-GGGGAGGGGCAACTGAAGGGGGGG-CCACA

Supplementary Table S3. Oligonucleotide sequences flanking PQS and no-PQS regions (ectopic regions).

**Upstream no-PQS regions (ectopic regions)**	**Sequence**	**Product length (bp)**
pJAZ8-fw	TATGGATGTGGATGCCGATG	174
pJAZ8-rv	AACTGTAGAATTTGGGCGCC	
pWRKY-fw	TGGCAGCGATTCCCAAATTG	149
pWRKY-rv	ACAGACTAGCTAGAGACCCCA	
pMAPK1-fw	GCCGCCAAAAGAAAATTGCG	195
pMAPK1-rv	GGGAGGGCTTAAGAAACCGA	
pACO1-fw	TGGCTAAAGGTGAATGCAAGG	183
pACO1-rv	GCTCTAAGGTTCCAAACATAGCT	
pAP2-EREBP	ACAAACCGAAGCACACGAGA	178
pAP2-EREBP	TGGGAGTAGACTGAACTGTTGT	
**PQS-Target Regions**		
JAZ8-G4fw	ATTTCATCGGCACAGTGGGC	172
JAZ8-G4rv	GTGCGGGTCATCGATAGGAC	
WRKY-G4fw	TGTCAACGTTTAACCCCTCGA	180
WRKY-G4rv	GAACCAAACCAGCAGCAGAG	
MAPK1-G4fw	CGCTCACTTTCTCGCTACCT	153
MAPK1-G4rv	CGTCAAAAGAGGAAGCGCAG	
ACO1-G4fw	GAGAATCCCGTGCGTGCAT	176
ACO1-G4rv	CGAGCTTTCCCGACTTGTCA	
AP2-EREBP-G4fw	GCAGGGAAGGTGGTTATGCT	147
AP2-EREBP-G4rv	AGAGCAGTAGACCAAGCGTG	

Supplementary Figures

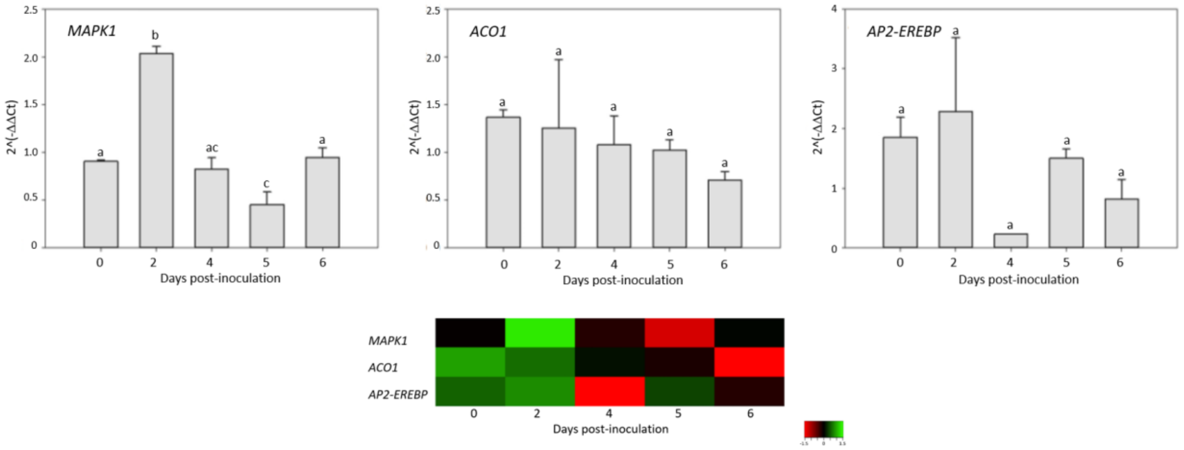

Supplementary Figure S1. Differential expression profiles of selected genes following inoculation with *T. atroviride*.

Relative expression levels of selected genes were determined by RT-qPCR between 0 and 6 days after inoculation with *T. atroviride*. Each bar graph represents the mean value of $2^{-\Delta\Delta Ct}$ ± standard deviation of three independent biological replicates across different time points post-*Trichoderma* inoculation. Genes analyzed include Mitogen-activated protein kinase 1 (*MAPK1*, GRMZM2G053987), 1-aminocyclopropane-1-carboxylate oxidase (*ACO1*, GRMZM2G007249), and Ethylene-responsive transcription factor (*AP2-EREBP*, GRMZM2G085678). The heatmap illustrates the differential expression profiles of the selected genes compared with uninoculated controls. The figure was generated using the Heatmapper web tool (http://www.heatmapper.ca/).

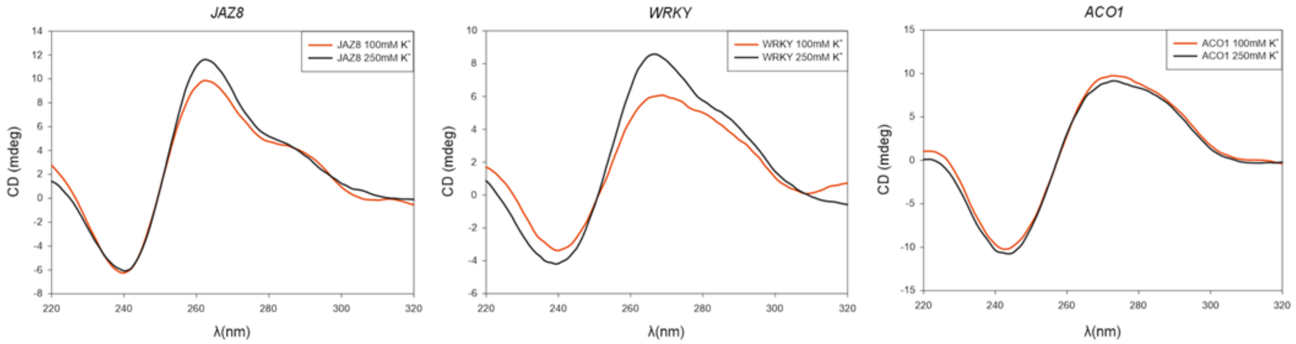

Supplementary Figure S2. CD spectra of oligonucleotides corresponding to the PQSs identified within the **PPRs** of *JAZ8*, *WRKY* and *ACO1* folded in the presence of 100 and 250 mM KCl**.**

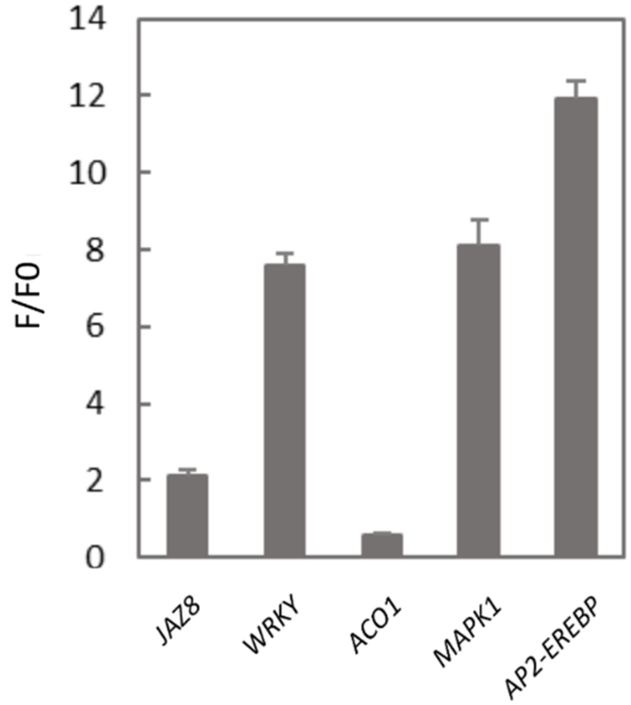

Supplementary Figure S3. ThT fluorescence assay for selected PQSs. Bar graph of fluorescence enhancement (F/F0) of ThT when exposed to selected PQSs folded in the presence of 100 mM K^+. Each bar represents the mean of three technical repeats and error bars correspond to standard deviation (SD). Considering a threshold of 5-fold fluorescence increase as G4 formation evidence, results indicate the formation of G4s by *MAPK1*, *WRKY* and *AP2-EREBP* PQSs, although a slight increase in fluorescence was observed for *JAZ8* PQS.
